# Intensive urate-lowering with pegloticase plus methotrexate co-therapy in uncontrolled gout patients with and without chronic kidney disease: A retrospective case series

**DOI:** 10.1097/MD.0000000000037424

**Published:** 2024-03-08

**Authors:** John Albert, Aaron Broadwell, Lissa Padnick-Silver, Brad Marder, Brian LaMoreaux

**Affiliations:** a Rheumatic Disease Center, Milwaukee, WI; b Rheumatology and Osteoporosis Specialists, Shreveport, LA; c Horizon Therapeutics plc (now Amgen, Inc.), Deerfield, IL.

**Keywords:** chronic kidney disease, gout, methotrexate, pegloticase, renal function

## Abstract

Chronic kidney disease (CKD) and gout commonly co-occur. Pegloticase lowers serum urate (SU) in uncontrolled gout patients but antidrug antibodies limit urate-lowering response and increase infusion reaction (IR) risk. Methotrexate (MTX) co-administration increases pegloticase response rate and mitigates IR risk but CKD limits MTX use. This pooled case series examined pegloticase + MTX co-therapy in uncontrolled gout patients with and without CKD. Cases of pegloticase + MTX co-therapy in existing datasets were retrospectively examined. Baseline eGFR classified patients as CKD (eGFR < 60 mL/min/1.73 m^2^) or non-CKD (eGFR ≥ 60 mL/min/1.73 m^2^). Patient characteristics, treatment parameters, laboratory values, urate-lowering response rate (≥12 pegloticase infusions received and SU < 6 mg/dL just before infusion 12), and AEs were examined. Fifteen CKD (eGFR: 43.2 ± 11.3 mL/min/1.73 m^2^; SU: 8.6 ± 2.2 mg/dL), 27 non-CKD (eGFR: 82.9 ± 19.0 mL/min/1.73 m^2^; SU: 9.5 ± 1.7 mg/dL) patients were included. Comorbidity profiles were similar, but CKD patients were older (72.0 ± 9.9 vs 52.3 ± 14.3 years) and more often female (33.3% vs 7.4%). Treatment parameters were similar with 4-week MTX Run-in followed by mean of 14.7 ± 8.1 [CKD] vs 14.1 ± 7.1 [non-CKD] pegloticase infusions. However, CKD patients had lower MTX dose (14.8 ± 5.8 vs 19.3 ± 4.9 mg/week). Urate-lowering response was similar (92% vs 86%). eGFR increased during treatment in 60% of CKD (+11.5 ± 20.9 mL/min/1.73 m^2^, 87% stable/improved CKD-stage) and 44% of non-CKD (+4.2 ± 15.0 mL/min/1.73 m^2^) patients. AEs were similar (≥1 AE CKD: 53%, non-CKD: 67%; gout flare most-reported). One case each of pancytopenia and IR (mild) occurred in non-CKD patients. These real-world data show similar pegloticase + MTX efficacy in CKD and non-CKD patients. No new safety signals were identified, with most CKD patients showing renal function stability or improvement during therapy.

**Key points**

- These real-world data show a high urate-lowering response rate to pegloticase + MTX co-therapy in both CKD and non-CKD patients.
- Pegloticase + MTX co-therapy was well-tolerated in patients with CKD, with no new safety signals identified.
- CKD patients in which methotrexate use was appropriate generally showed renal function stability or improvement during pegloticase + MTX therapy.

## 1. Introduction

The relationship between chronic kidney disease (CKD) and gout is bidirectional, with gout patients having an increased risk for CKD^[1–4]^ and CKD patients an increased risk for gout.^[5–7]^ Further, hyperuricemia has been shown to be an independent risk factor for CKD worsening,^[8,9]^ particularly in women.^[9]^ Because CKD is prevalent in gout patients, renal function is an important factor in managing uncontrolled gout, particularly with respect to medication dosing, drug toxicity, and drug/drug interactions.

Pegloticase, a recombinant PEGylated uricase, can rapidly decrease serum urate levels (SU) in patients refractory to, intolerant of, or with contraindications to oral urate-lowering therapies.^[10]^ However, many patients administered pegloticase monotherapy develop antidrug antibodies against pegloticase, which have been associated with loss of urate-lowering response and infusion reactions (IRs).^[10,11]^ The literature now widely supports coadministration of an immunomodulator with pegloticase,^[12]^ with the MIRROR randomized, controlled trial (MIRROR RCT) confirming the safety and efficacy superiority of pegloticase + methotrexate (MTX) co-therapy over pegloticase monotherapy. This trial demonstrated a urate-lowering response rate during treatment month 6 of 71% vs 39% and an IR rate of 4% vs 31% in the pegloticase + MTX vs pegloticase + placebo treatment groups.^[13]^ However, caution is needed when using MTX in patients with renal impairment because of tolerability and toxicity concerns.^[14]^

Though randomized clinical trials examining pegloticase plus immunomodulation co-therapy included some patients with an eGFR between 40 and 60 mL/min/1.73 m^2^, data in patients with comorbid gout and advanced CKD is limited. However, several published case series describing real-world pegloticase + MTX use have included patients with pre-therapy eGFR < 60 mL/min/1.73 m^2^. The current study pooled and examined available case data previously collected for other studies^[15–17]^ to further examine treatment outcomes and renal function during pegloticase + MTX co-treatment in patients with and without CKD at baseline.

## 2. Methods

This retrospective study examined deidentified real-world case data previously collected for prior retrospective study.^[15–17]^ The Western IRB (Puyallup, WA) had reviewed each of those studies, assigning all exempt status. Because all data was existing and de-identified, this study did not meet the definition of human subjects research (NIH tool, https://grants.nih.gov/policy/humansubjects/research.htm) and further IRB review/approval was not needed.

All patients who underwent pegloticase + MTX co-therapy were included and categorized as CKD (eGFR < 60 mL/min/1.73 m^2^) or non-CKD (eGFR ≥ 60 mL/min/1.73 m^2^) based on pretreatment (baseline) eGFR. eGFR was calculated from serum creatinine using the MDRD equation.^[18]^ As part of routine care for pegloticase and MTX use, all patients had SU, renal function, blood cell counts, and liver function closely monitored during therapy. Patient characteristics, pegloticase treatment parameters, proportion of treatment responders, renal function changes (eGFR, CKD stage), and adverse events, as noted in the medical record, were examined. CKD stage was defined using standard eGFR-based categorization. Treatment response was defined as ≥ 12 pegloticase infusions received with an SU < 6 mg/dL just prior to infusion 12 (ongoing patients with < 12 infusions were excluded from responder rate analyses).

Data are presented as mean (±SD) or n (%) as appropriate. Differences in parameters were statistically examined using Student *t* tests for continuous variables and Fisher exact tests for categorical ones. Potential factors that influenced eGFR changes during therapy were examined using descriptive statistics and linear regression analysis. Missing data were excluded from analyses. Statistical significance was defined as a two-tailed *P* < .05.

## 3. Results

### 3.1. Patients

A total of 42 uncontrolled gout patients treated with pegloticase + MTX co-therapy were identified (Table 1). Average patient age was approximately 60 years and most patients were male (83.3%) and Caucasian (81.0%). On average, patients had a 15.2 ± 13.1 year gout history (median: 11 years) and a pre-therapy SU of 9.2 ± 1.9 mg/dL. Nearly all patients (92.9%) had visible (subcutaneous) tophi.

**Table 1:** Pretreatment characteristics of uncontrolled gout patients with and without baseline CKD (pre-therapy eGFR < 60 mL/min/1.73 m^2^) who underwent pegloticase plus methotrexate co-therapy.

	All patients (N = 42)	Non-CKD (n = 27)	CKD (n = 15)	*P*-value* (CKD vs non-CKD)
Male, n (%)	35 (83.3%)	25 (92.6%)	10 (66.7%)	.003
Patient age, years, mean ± SD	59.3 ± 16.0	52.3 ± 14.3	72.0 ± 9.9	<.001
≥65 years	14 (33.3%)	5 (18.5%)	9 (60.0%)	.015
Race, n (%)				.833
Caucasian	34 (81.0%)	21 (77.8%)	13 (86.7%)	
Asian	5 (11.9%)	4 (14.8%)	1 (6.7%)	
African-American	3 (7.1%)	2 (7.4%)	1 (6.7%)	
Current smoker	9 (21.4%)	6 (22.2%)	3 (20.0%)	>.999
BMI, kg/m^2^, mean ± SD	33.8 ± 8.3	34.3 ± 7.9	33.0 ± 9.2	.655
Gout characteristics				
Gout duration†, years, mean ± SD	15.2 ± 13.1	13.0 ± 10.4	19.1 ± 16.6	.263
Pre-therapy SU, mg/dL, mean ± SD	9.2 ± 1.9	9.5 ± 1.7	8.5 ± 2.2	.146
Visible (subcutaneous) tophi, n(%)	39 (92.9%)	26 (96.3%)	13 (86.7%)	.287
Comorbidities				
Hypertension	33 (78.6%)	21 (77.8%)	12 (80.0%)	>.999
Obesity	22 (52.4%)	15 (55.6%)	7 (46.7%)	.749
Cardiovascular disease	13 (31.0%)	7 (25.9%)	6 (40.0%)	.488
Congestive heart failure	4 (9.5%)	2 (7.4%)	2 (13.3%)	.608
Coronary arterial disease	7 (16.7%)	4 (14.8%)	3 (20.0%)	.686
Atrial fibrillation	4 (9.5%)	4 (14.8%)	0	.279
Diabetes mellitus	12 (28.6%)	6 (22.2%)	6 (40.0%)	.292
Osteoarthritis	27 (64.3%)	18 (66.7%)	9 (60.0%)	.743
Hyperlipidemia/dyslipidemia	5 (11.9%)	2 (7.4%)	3 (20.0%)	.330
Renal characteristics				
eGFR, mL/min/1.73 m^2^, mean ± SD	68.7 ± 25.4	82.9 ± 19.0	43.2 ± 11.3	<.001
CKD stage (based on eGFR)				–
Stage 1	9 (21.4%)	9 (33.3%)	–	
Stage 2	18 (42.9%)	18 (66.7%)	–	
Stage 3a	9 (21.4%)	–	9 (60.0%)	
Stage 3b	4 (9.5%)	–	4 (26.7%)	
Stage 4	2 (4.8%)	–	2 (13.3%)	

BMI = body mass index, CKD = chronic kidney disease, eGFR = estimated glomerular filtration rate (MDRD equation^[18]^), SU = serum urate.

* Two-tailed *P*-values calculated using unpaired *t*-tests for continuous parameters and Fisher exact tests for categorical parameters.

† Unknown in 5 non-CKD, 1 CKD patients; noted as “long time” or “many” in 1 non-CKD, 2 CKD patients (N = 21, 12). missing values.

Fifteen patients (35.7%) had a baseline eGFR < 60 mL/min/1.73 m^2^ and were classified as having CKD; 27 patients (64.3%) had a baseline eGFR ≥ 60 mL/min/1.73 m^2^ and were classified as non-CKD. Patient characteristics and comorbidity profiles were similar, but those with CKD were older (72.0 ± 9.9 vs 52.3 ± 14.3 years, *P* < .001) and more often female (33.3% vs 7.4%, *P* = .003; Table 1).

### 3.2. Pegloticase plus methotrexate co-therapy

Treatment parameters were similar between CKD and non-CKD groups (Fig. 2A). MTX was initiated approximately 4 weeks before first pegloticase infusion in both groups, but the mean overall MTX dose was significantly lower in CKD patients (14.8 ± 5.8 vs 19.3 ± 4.9 mg/week, *P* = .019; includes both oral and subQ MTX). On average, the number of pegloticase infusions (CKD: 14.7 ± 8.1 vs non-CKD: 14.1 ± 7.1 infusions) and duration of pegloticase treatment (28.5 ± 17.1 vs 27.9 ± 15.1 weeks) were also similar. Further, a similar proportion of CKD and non-CKD patients had a sustained urate-lowering response through at least infusion 12 (91.7% [11/12] vs 86.4% [19/22]).

**Figure 1.**
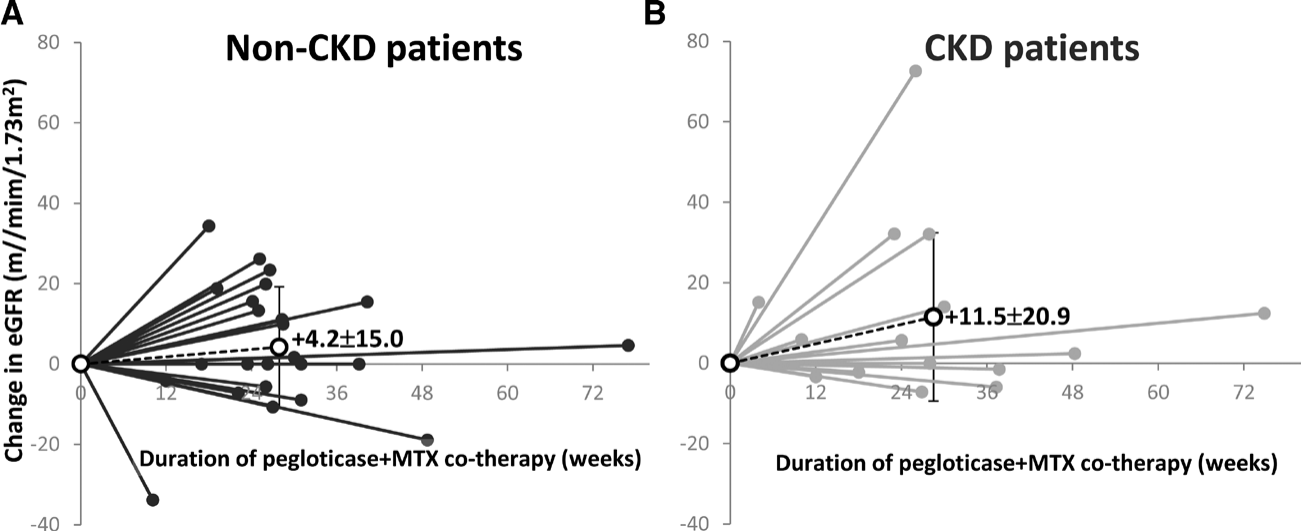
Change in eGFR during pegloticase + MTX co-therapy in non-CKD (A) and CKD (eGFR < 60 mL/min/1.73 m^2^, (B) patients. Mean therapy duration (time between first and last pegloticase infusion) was 28 weeks in both groups. eGFR was calculated from serum creatinine using the MDRD equation.^[18]^ Solid dots represent individual patient data. Open circles represent mean eGFR change over mean therapy duration. Error bars represent standard deviation. CKD = chronic kidney disease; MTX = methotrexate.

**Figure 2.**
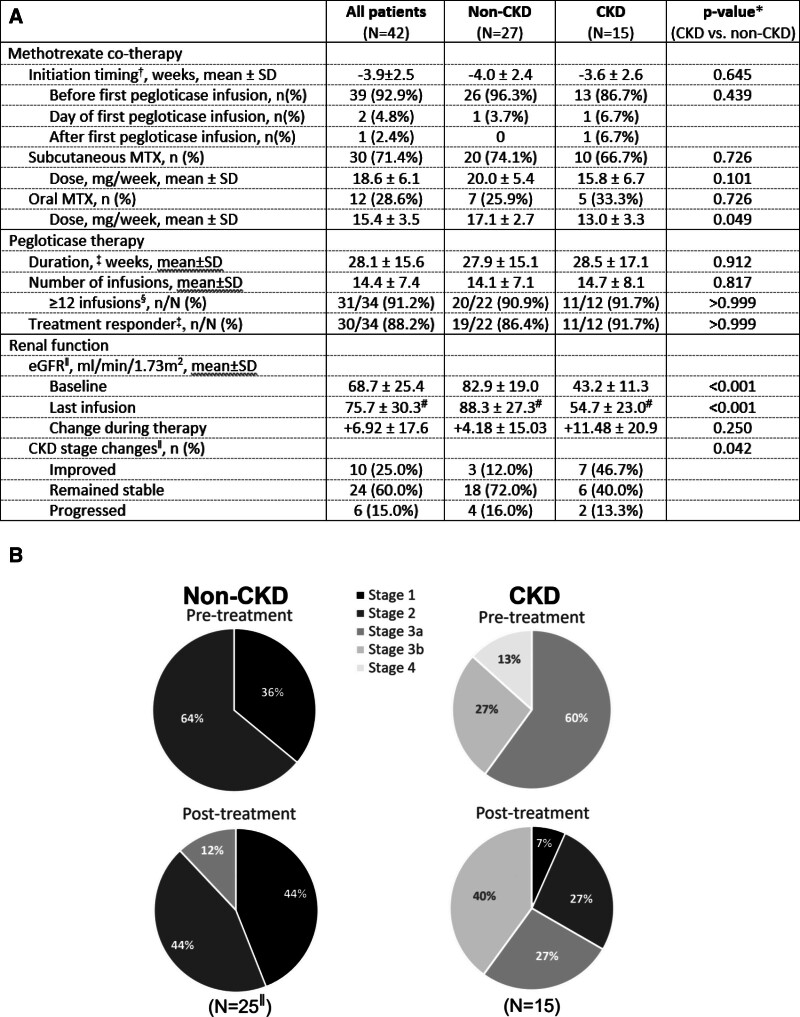
Pegloticase plus methotrexate treatment parameters (A) and changes in kidney function during treatment (A, B [darker shade indicates higher kidney function]). Of note, the 2 patients with pre-therapy stage 4 CKD improved to stage 3a CKD by the end of therapy (23 and 28 weeks). eGFR calculated using the MDRD equation.^[18]^ *Two-tailed *P*-values calculated using unpaired *t*-tests for continuous parameters and Fisher exact tests for categorical parameters. ^†^With respect to first pegloticase infusion, negative value indicates BEFORE first infusion. ^‡^Time between first and last pegloticase infusion. ^§^Excludes patients with ongoing therapy who had not yet reached infusion 12 (5 non-CKD, 3 CKD). ^‖^excludes 2 non-CKD patients with missing post-therapy eGFR measurement (all patients N = 40, non-CKD N = 25). ^#^Compared to pre-therapy eGFR: all patients *P* = .017, non-CKD *P* = .177, CKD *P* = .052.

### 3.3. Change in kidney function during pegloticase plus methotrexate co-therapy

On average, kidney function slightly improved during therapy. Baseline (pretreatment) eGFR averaged 68.7 ± 25.4 mL/min/1.73 m^2^, with 9 patients (21.4%) having Stage 3a CKD, 4 patients (9.5%) having Stage 3b CKD, and 2 patients (4.8%) having Stage 4 CKD. At last pegloticase infusion, eGFR averaged 75.7 ± 30.3 mL/min/1.73 m^2^ with a mean change from baseline of +6.9 ± 17.6 mL/min/1.73 m^2^ (*P* = .017 compared to a zero change). Further, CKD stage was stable or improved in 85.0% (34/40) of patients (Fig. 2).

Findings were similar in patients with and without pretreatment CKD. In the non-CKD group, post-therapy eGFR did not significantly differ before and after therapy (84.1 ± 19.1 vs 88.3 ± 27.3 mL/min/1.73 m^2^; *P* = .177, N = 25 [2 missing post-therapy values]; Fig. 1). Twenty-one of 25 patients (84.0%) had improved or stable CKD stage (Fig. 2). Of the remaining 4 patients, 1 was categorized as Stage 1 CKD (eGFR = 90.9 ml/min/1.73m^2^) before treatment and Stage 2 CKD (eGFR = 86.7 mL/min/1.73 m^2^) after treatment; 3 had Stage 2 CKD prior to treatment and Stage 3a CKD at the end of treatment. In the CKD group, post-therapy eGFR tended to be higher than pre-therapy values (43.2 ± 11.3 vs 54.7 ± 23.0 mL/min/1.73 m^2^, *P* = .052; change in eGFR: +11.5 ± 20.9; Fig. 1). Thirteen of 15 patients (86.7%) had improved or stable CKD stage (Fig. 2). The remaining 2 patients had Stage 3a CKD prior to therapy and had progressed to Stage 3b after therapy. Of note, 9 CKD patients (60%) had an increase in eGFR during therapy, including 2 patients with stage 4 CKD at baseline who both improved to Stage 3a (therapy duration of 23 and 28 weeks [12 and 14 infusions]). Additionally, 1 patient with a 2-year history of gout had a marked, progressive improvement in kidney function (pre-therapy vs post-therapy eGFR: 51.3 vs 123.9 mL/min/1.73 m^2^, serum creatinine: 1.44 vs 0.67 mg/dL; therapy duration of 26 weeks [14 infusions]). The patient also had a history of hypertension, osteoarthritis, obesity, thyroid disease, and cancer.

### 3.4. Factors related to eGFR change during pegloticase plus methotrexate co-therapy

Patients with increased/stable eGFR and decreased eGFR during pegloticase + MTX co-therapy were compared to identify potential factors that may have influenced eGFR changes during pegloticase + MTX co-therapy. Patient and treatment characteristics were statistically similar between groups. However, patients who had a decrease in eGFR during pegloticase + MTX treatment tended to have a lower baseline renal function (62.0 ± 21.0 vs 71.7 ± 27.7 mL/min/1.73 m^2^) and longer gout duration (20.8 ± 12.3 vs 13.1 ± 13.0 years, Table 2) than those with a change of ≥0. Univariate linear regression showed that the relationship between change in eGFR and baseline eGFR was not significant (R^2^ = .0047, *P* = .674) but the relationship between change in eGFR and gout duration was (R^2^ = .1253, *P* = .043). Further, when patients were divided by median gout duration (11 years), those with longer (>11 years) vs shorter (≤11 years) gout had a significantly lower eGFR change (+0.7 ± 15.5 vs +14.5 ± 19.6 mL/min/1.73 m^2^, *P* = .032) and were more likely to have a decrease in eGFR (46.7% vs 11.1%; OR [95% CI]: 7.00 [1.17–41.76], *P* = .033) during therapy. Though not statistically significant, patients with a change in eGFR < 0, tended to have higher prevalences of cardiovascular disease (33.3% vs 14.3%) and diabetes (41.7% vs 21.4%, Table 2).

**Table 2:** Patient and treatment characteristics in those with and without stable/increased eGFR during pegloticase + MTX co-therapy.

	Change in eGFR ≥ 0 (N = 28)	Change in eGFR < 0 (N = 12)	*P*-value
Patient characteristics			
Male, n(%)	23 (82.1%)	10 (83.3%)	>.999
Age, years, mean ± SD	59.5 ± 17.2	59.5 ± 15.0	>.999
Caucasian, n(%)	21 (75.0%)	11 (91.7%)	.396
BMI, kg/m^2^, mean ± SD	34.0 ± 8.7	34.6 ± 8.0	.837
Current smoker, n(%) (N = 25, 12)	6 (24.0%)	3 (25.0%)	>.999
Gout characteristics			
Gout duration*, years, mean ± SD	13.1 ± 13.0	20.8 ± 12.3	.137
Pre-therapy SU, mg/dL, mean ± SD	9.0 ± 2.0	9.5 ± 1.9	.441
Visible (subcutaneous) tophi, n(%)	25 (89.3%)	12 (100%)	.541
Comorbidities			
Hypertension	22 (78.6%)	10 (83.3%)	>.999
Obesity	15 (53.6%)	7 (58.3%)	>.999
Cardiovascular disease	4 (14.3%)	4 (33.3%)	.188
Diabetes mellitus	6 (21.4%)	5 (41.7%)	.254
Osteoarthritis	16 (57.1%)	9 (75.0%)	.477
Hyperlipidemia/dyslipidemia	4 (14.3%)	1 (8.3%)	>.999
Renal characteristics			
eGFR, ml/min/1.73 m^2^, mean ± SD	71.7 ± 27.7	62.0 ± 21.0	.239
Change in eGFR during therapy	+13.8 ± 15.8	-9.1 ± 9.1	<.001
CKD Stage (based on baseline eGFR)			.354
Stage 1	8 (28.6%)	1 (8.3%)	
Stage 2	10 (35.7%)	6 (50.0%)	
Stage 3a	7 (25.0%)	3 (25.0%)	
Stage 3b	1 (3.6%)	2 (16.7%)	
Stage 4	2 (7.1%)	0	
Treatment parameters			
MTX co-therapy			
Time initiated, weeks, mean ± SD	-28.8 ± 16.2	-25.8 ± 20.5	.651
Oral MTX, n(%)	9 (32.1%)	3 (25.0%)	.725
Dose, mg/week, mean ± SD	16.3 ± 2.5	18.3 ± 2.9	.124
Subcutaneous MTX, n(%)	19 (67.9%)	9 (75.0%)	.725
Dose, mg/week, mean ± SD	18.2 ± 5.8	18.1 ± 6.8	.970
Pegloticase infusions, mean ± SD	15.5 ± 7.8	13.3 ± 5.7	.333
Treatment duration, weeks, mean ± SD	30.7 ± 16.5	25.7 ± 11.8	.292
Treatment responder†, n (%)	22/23 (95.7%)	8/9 (88.9%)	>.999

MTX = methotrexate, SU = serum urate.

* Excludes patients with an unknown gout duration (N = 24, 9).

† Treatment responder defined as ≥12 pegloticase infusions received with SU < 6 mg/dL just prior to infusion 12 (excludes patients with ongoing therapy who had not yet reached infusion 12).

### 3.5. Adverse events

Adverse events were similarly noted in patients with and without baseline CKD, with at least 1 adverse event reported in 53.3% (8/15) and 66.7% (18/27) of patients, respectively (Table 3). Gout flare was the most common adverse event in both groups (CKD: 7/15 [46.7%], non-CKD: 11/27 [40.7%]). CKD stage progression was next common, with 1 non-CKD patient moving from Stage 1 to Stage 2, 3 non-CKD patients moving from Stage 2 to 3a, and 2 CKD patients moving from Stage 3a to 3b. Two non-CKD patients experienced LFT elevations likely related to alcohol use, with possible relation to MTX in 1 patient as fully described elsewhere.^[15]^ Stroke (deemed unrelated to pegloticase or MTX), pancytopenia, and a mild IR were also noted in 1 non-CKD patient each. As previously described,^[15]^ the pancytopenia was deemed related to MTX use and resulted in MTX discontinuation. The patient received the last 4 of 13 pegloticase doses without MTX co-administration and met study criteria for treatment response.

**Table 3:** Adverse events observed in CKD and non-CKD patients undergoing pegloticase + MTX co-therapy.

	Non-CKD (N = 27 patients)	CKD (N = 15 patients)
≥1 adverse event reported, n (%)	18 (66.7%)	8 (53.3%)
Acute gout flare	11 (40.7%)	7 (46.7%)
Number flares/patient with ≥1 flare	2.2 ± 1.8	1.4 ± 0.8
CKD development/worsening*	4 (16.0%)	2 (13.3%)
New extremity pain	2 (7.4%)	1 (6.7%)
Elevated LFTs	2 (7.4%)	0
Transient rise in serum creatinine	1 (3.7%)	0
Pancytopenia	1 (3.7%)	0
Infusion reaction	1 (3.7%)	0
Stroke	1 (3.7%)	0
Flare severity	(N = 24 flares)	(N = 10 flares)
Mild, n (% flares)	6 (29%)	6 (60%)
Moderate, n (% flares)	4 (19%)	2 (20%)
Severe, n (% flares)	11 (52%)	2 (20%)

MTX = methotrexate.

* Represents pre-/post-therapy CKD stage increase (includes 1 non-CKD patient who moved from stage 1 to 2 based on eGFR).

## 4. Discussion

This retrospective real-world review of a limited number of cases begins to examine pegloticase + MTX co-therapy in CKD patients, with 40% of included CKD patients having Stage 3b or higher disease. Pegloticase + MTX co-therapy resulted in similar rates of sustained SU-lowering in uncontrolled gout patients with and without CKD (91.7% vs 86.4%). This finding is in agreement with a prior study showing similar pegloticase monotherapy efficacy in patients regardless of pretreatment CKD stage.^[19]^

The literature strongly supports the use of immunomodulation as co-therapy to pegloticase to increase treatment response rates^[12]^ and lower the risk of IRs.^[20]^ Immunomodulatory agents reported in the literature include MTX,^[13,15,21,22]^ mycophenolate mofetil,^[23]^ leflunomide,^[17]^ and azathrioprine,^[24]^ with MTX most studied and now recommended in the updated prescribing information as co-therapy to pegloticase.^[25]^ However, MTX is cleared by the kidneys and must be used with caution in CKD patients, with careful monitoring for known MTX toxicities (e.g., liver function test elevations, bone marrow suppression^[14]^). As a result, MIRROR RCT, which directly compared pegloticase + MTX and pegloticase + placebo co-therapy, excluded patients with a baseline eGFR < 40 mL/min/1.73 m^2^.^[13]^ In the current study, adverse events were noted with similar frequency in patients with CKD compared to those without CKD, including those most commonly associated with MTX.^[14]^ However, other immunomodulatory agents may be better suited for some CKD patients, but their use as co-therapy to pegloticase has not yet been fully vetted in large clinical trials.

Close monitoring of renal function in the current study indicated CKD stability or improvement during therapy in 85.0% of all treated patients and 86.7% of those with CKD (mean treatment duration approximately 28 weeks). Renal function stability has been previously reported during pegloticase monotherapy in patients with and without CKD,^[19]^ but the literature on renal function during pegloticase plus immunomodulator co-therapy is limited. Preliminary findings of the MIRROR RCT trial examining the safety and efficacy of MTX vs placebo as co-therapy to pegloticase showed renal function stability after 24 weeks^[26]^ and 52 weeks^[27,28]^ of therapy in both the pegloticase + MTX and pegloticase + placebo treatment groups. Renal function changes during pegloticase administered with other immunomodulatory agents have not yet been reported.

Factors potentially influencing eGFR changes were examined by comparing patients with and without an eGFR decrease during therapy. Two potential factors were identified, including baseline eGFR and gout duration. However, in this small group of patients, univariate analysis revealed that only gout duration significantly influenced the change in eGFR. Patients with gout of longer duration patients (>11 years) were 7 times more likely to have an eGFR decline during therapy than shorter duration patients (46.7% vs 11.1%, OR: 7.00).

This study had several limitations. First, though relatively large for a retrospective medical record review case series, the study sample overall was small and further study on a larger group of patients with CKD is needed. Second, as common in observational studies, treatment duration and monitoring timepoints were not standardized. Future prospective studies specifically examining pegloticase therapy in CKD patients would overcome this issue. Third, retrospective chart reviews are susceptible to a selection bias. However, each study based patient selection on pegloticase treatment during a predefined time period and all eligible patients were included.

In conclusion, this retrospective chart review study of uncontrolled gout patients provides valuable information on pegloticase + MTX co-therapy in patients with and without CKD. Pretreatment CKD did not influence pegloticase urate-lowering response rate or adverse event occurrence. Of note, CKD stage remained stable or even improved in more than 85% of patients, with little-to-no influence of baseline eGFR on eGFR changes during therapy. However, patients with a shorter gout duration (≤11 years) were more likely to have eGFR stability/improvement than those with longer disease. This finding suggests potential sparing of gout-related renal function loss with earlier uncontrolled gout management. Overall study findings provide much needed insight into renal function changes during pegloticase + MTX co-therapy in uncontrolled gout patients with and without CKD.

## Acknowledgments

We thank Qianhong Fu, MS, employee of and stockholder in Horizon (now Amgen, Inc.), for statistical consultation.

## Author contributions

**Conceptualization:** John Albert, Aaron Broadwell, Lissa Padnick-Silver, Brad Marder, Brian LaMoreaux.

**Data curation:** John Albert, Aaron Broadwell.

**Formal analysis:** Lissa Padnick-Silver.

**Funding acquisition:** Brian LaMoreaux.

**Investigation:** John Albert, Aaron Broadwell, Lissa Padnick-Silver.

**Methodology:** John Albert, Aaron Broadwell, Lissa Padnick-Silver, Brad Marder, Brian LaMoreaux.

**Project administration:** Brian LaMoreaux.

**Resources:** Brian LaMoreaux.

**Supervision:** John Albert, Aaron Broadwell, Brad Marder, Brian LaMoreaux.

**Validation:** Lissa Padnick-Silver.

**Visualization:** Lissa Padnick-Silver.

**Writing – review & editing:** John Albert, Aaron Broadwell, Lissa Padnick-Silver, Brad Marder, Brian LaMoreaux.

**Writing – original draft:** Lissa Padnick-Silver.
